# Reduced Muscle Loss in Patients With NSCLC Taking Fibrates: Findings From a Retrospective Observational Study

**DOI:** 10.1002/jcsm.70016

**Published:** 2025-08-20

**Authors:** Rahmi Elahjji, Tahj Blow, Rahul Grover, Maurice A. Hurd, Richard F. Dunne, Bette J. Caan, Elizabeth M. Cespedes Feliciano, Andrew J. Plodkowski, James H. Flory, Marcus D. Goncalves

**Affiliations:** ^1^ Weill Cornell Medicine New York New York USA; ^2^ University of Rochester Medical Center Rochester New York USA; ^3^ Kaiser Permanente Oakland California USA; ^4^ Memorial Sloan Kettering Cancer Center New York New York USA

**Keywords:** cachexia, fibrates, lung cancer, PPAR‐α, skeletal muscle

## Abstract

**Background:**

The cancer‐anorexia‐cachexia syndrome (CACS) is a common and debilitating wasting disorder characterized by loss of skeletal muscle and worse morbidity and mortality. In pre‐clinical studies, CACS is associated with loss of peroxisome proliferator‐activated receptor alpha (PPAR‐α) dependent ketone production in the liver. Fibrates are PPAR‐α agonists that are commonly used to treat dyslipidemia. Treating mice with fibrates was found to prevent skeletal muscle loss. We examine whether patients with cancer treated with PPAR‐α agonists experience less CACS.

**Methods:**

We performed a retrospective cohort study of patients (*N* = 6922) at Memorial Sloan Kettering Cancer Center who were diagnosed with non‐small cell lung cancer (NSCLC) between 2002 and 2017 and were incidentally prescribed fenofibrate or gemfibrozil at the time of diagnosis. These patients were compared to a propensity score‐matched control set who were not taking either drug. The primary outcome included a composite outcome of CACS, which included significant weight loss before or after the time of diagnosis. Secondary outcomes included change in cross‐sectional skeletal muscle area over time as measured in serial CT imaging studies and overall survival. Descriptive statistics, Kaplan–Meier analysis and multivariable logistic regression were performed to compare outcomes between the two groups.

**Results:**

Among patients with NSCLC, 149 were taking fenofibrate or gemfibrozil at the time of diagnosis. A 2:1 propensity score‐matched cohort of 298 patients was created that was well‐matched with regard to baseline characteristics. Regarding the primary composite outcome, there was no significant difference in the prevalence of CACS between those taking fibrates and propensity‐matched controls (49.7 vs. 46.6%). When skeletal muscle mass was measured directly using cross‐sectional imaging, patients on fibrates were found to have lost significantly less muscle area over time (−3.3 vs.−4.2%, *p* = 0.03). There was no difference in overall survival between groups.

**Conclusion:**

Patients with NSCLC taking fibrates at the time of diagnosis lost less muscle area over time. In a secondary analysis, this change was not associated with a change in overall survival, though this study was likely underpowered for this analysis.

## 1 Introduction

The cancer‐anorexia‐cachexia syndrome (CACS) is a systemic metabolic disorder characterized by the catabolism of nutrient‐rich tissues in the setting of cancer. Key features include loss of overall body weight, reduced food intake, increased markers of systemic inflammation and progressive functional impairment [1]. Poor clinical outcomes of CACS include poorer tolerance to anti‐neoplastic therapy, a reduction in quality of life, and decreased overall survival [2].

Non‐small cell lung cancer (NSCLC) is the 2nd most common type of cancer in the United States and has the highest mortality of all cancers [3]. Patients with NSCLC have a high prevalence of CACS both at the time of diagnosis (50%) and in the setting of advanced disease (50%–75%) [4]. Further, patients with NSCLC often present with advanced disease and often require a combination of multimodal therapy as well as palliative care.

There is no widely accepted therapy to prevent or treat CACS. However, an evolving body of recent research has attempted to elucidate biological mechanisms underlying the pathophysiology of CACS, with the goal of identifying mechanistic targets for potential therapies. New evidence of local inflammatory and metabolic mediators in the tumour microenvironment interacting with the endocrine, metabolic and nervous systems has been implicated in the pathogenesis of CACS [5]. Furthermore, improved animal models, along with new genomic and metabolomic techniques, have been better able to replicate CACS in the setting of different host tumours [6].

We have previously shown that mice with lung cancer‐induced CACS develop a distinct metabolic signature that features loss of peroxisome proliferator‐activated receptor alpha (PPAR‐α) dependent ketone production in the liver, and atrophy of skeletal muscle fibres. Treatment of these mice with the PPAR‐α agonist, fenofibrate, restored hepatic ketone production and prevented skeletal muscle atrophy and weight loss [7]. Fibrates are widely prescribed medications for hypertriglyceridemia. We hypothesized that patients with NSCLC who were incidentally on a fibrate medication at the time of diagnosis would have less skeletal muscle loss and less weight loss over time.

## 2 Methods

### 2.1 Design

We performed a retrospective cohort study of adult patients 18 years and older with confirmed pathologic diagnoses of NSCLC between 1/1/2002 and 12/31/2017 at Memorial Sloan Kettering Cancer Center (MSKCC), a specialty referral cancer center (New York, New York). Identification of study patients and data abstraction was conducted with the DataLine service, an automated data abstraction service of existing EHR data, which was supplemented by manual abstraction of clinical variables when necessary. The study was approved by the institutional review board.

### 2.2 Patient Population

We evaluated all patients. We excluded patients with significant time (>90 days) between their pathologic diagnosis and their initial visit at MSKCC. We also excluded patients with synchronous non‐pulmonary tumours. We identified patients who were documented in their outpatient medical record to be taking either fenofibrate or gemfibrozil within 90 days of their initial diagnosis of NSCLC. We included patients who were documented to be taking either generic or brand names of these medications at any dose at the time of diagnosis. Patients documented not to be taking fibrates within 90 days of their diagnosis were identified as the initial control cohort.

Baseline characteristics about each patient cohort including age at diagnosis, sex, year of diagnosis, smoking status, Charlson Comorbidity Index (CCI), tumour histology sub‐type and American Joint Committee on Cancer (AJCC) summary staging were collected.

Additional clinical, tumour, treatment and radiologic data was collected, including the type of treatment ever received (chemotherapy, immunotherapy, targeted therapy, radiation therapy, or cancer directed surgery) and tumour mutation status (KRAS, EGFR, TP53). For all patients, the date of diagnosis, date of death and/or date of last follow‐up was also documented. For anthropometric measurements, we recorded the height and weight at the time of diagnosis (within 30 days).

### 2.3 Outcomes

The primary outcome was a composite outcome of cachexia which included patient report of significant weight loss at the time of diagnosis (defined as the patient's subjective endorsement of ‘clinically significant weight loss’ defined as greater than > 5% weight loss in the preceding 6 months), > 5% weight loss from the date of diagnosis to 6 months later (within 30 days), or > 2% weight loss from the date of diagnosis to 6 months later and any BMI < 20 kg/m^2^. These parameters were based on previously described international consensus definitions for cancer cachexia [1]. We recorded height and weight six when available from clinical data 6 months after diagnosis (within 30 days) and compared to baseline height and weight to calculate change in weight.

Change in cross‐sectional skeletal muscle area on imaging was included as a secondary outcome. We reviewed all available imaging data for all patients and identified patients with available CT Chest imaging performed within 30 days of diagnosis and 6 months after diagnosis (within 30 days). In this subset of patients, cross‐sectional skeletal muscle area was measured through a semi‐automated previously validated technique by one reader who was trained and supervised by a consulting radiologist [8, 9, 10]. Using iNtuition software (TeraRecon, Houston, TX, USA), skeletal muscle cross‐sectional area was measured at the T12 vertebral level by including mean attenuation values between −29 and 150 HU and manually excluding non‐muscular soft tissues. As previously described, analysis of thoracic skeletal muscle has been found to correlate relatively well with whole body skeletal muscle (*R* ^2^ = 0.78). The percent change in cross‐sectional skeletal muscle area was considered to reduce the influence of variation from study technique and baseline variability.

As an additional secondary outcome, overall survival was defined as the time from the date of diagnosis until either the date of last follow‐up or the date of death.

### 2.4 Statistical Analysis

Standard descriptive statistics, t‐test for continuous variables and chi‐square or Fisher's exact test for categorical variables were used to determine differences in baseline characteristics between both groups. A propensity‐score analysis was performed to balance the distribution of confounding variables between the fibrates cohort and the initial control cohort. Propensity score matching can reduce bias in observational studies by balancing the distribution of measured baseline confounding variables between groups [11]. Patients were matched based on their baseline covariates in a 2:1 ratio without replacement. Propensity score matching was performed using the covariates age, sex, year of diagnosis, smoking status, tumour histology and tumour summary staging. Patients were matched in a 2:1 ratio using the nearest neighbour method without replacement. Outcomes for the propensity‐score matched groups were compared with t‐test, chi‐square test and logistic regression to adjust for potential confounding variables. Overall survival was estimated by Kaplan–Meier analysis and compared with log‐rank test.

A two‐sided *p*‐value of < 0.05 was considered statistically significant. Analysis was conducted using the R statistical software (http://www.r‐project.org/).

## 3 Results

Among 6922 patients who were diagnosed with NSCLC between January 1st, 2002 and December 31st, 2017, 149 were documented to be taking either fenofibrate or gemfibrozil in their outpatient medical record. Baseline characteristics of both patients taking fibrates and control patients are summarized in Table 1. Prior to propensity‐score matching, statistically significant baseline differences between fibrates and control patients included age (69.4 vs. 66.0), sex (61.7% male vs. 45.7% male), year of tumour diagnosis, tumour histology and tumour summary staging.

**TABLE 1:** Baseline characteristics.

	Fibrates	Control	*p*
N	149	6773	
Age (mean (SD))	69.42 (7.60)	66.02 (11.03)	**< 0.001**
Sex = male N (%)	92 (61.7)	3093 (45.7)	**< 0.001**
Year of tumour diagnosis N (%)			**0.003**
2002–2006	21 (14.1)	1703 (25.1)	
2007–2011	63 (42.3)	2170 (32.0)	
2012–2017	65 (43.6)	2900 (42.8)	
CCI (mean (SD))	4.89 (2.75)	5.06 (2.20)	0.37
Smoking status N (%)			0.47
Never	21 (14.1)	1182 (17.5)	
Smoker	128 (85.9)	5575 (82.3)	
Unknown	0 (0.0)	16 (0.2)	
Histology N (%)			**0.001**
Adenocarcinoma	107 (71.8)	4980 (73.5)	
Large cell	3 (2.0)	101 (1.5)	
Other	4 (2.7)	691 (10.2)	
Squamous	35 (23.5)	1001 (14.8)	
Summary stage N (%)			**< 0.001**
Localized	68 (45.6)	1094 (16.2)	
Regional both	15 (10.1)	529 (7.8)	
Regional extension	13 (8.7)	362 (5.3)	
Regional nodes	16 (10.7)	764 (11.3)	
Regional nos	0 (0.0)	4 (0.1)	
Distant	37 (24.8)	4009 (59.2)	
Unstaged	0 (0.0)	11 (0.2)	

Abbreviations: CCI = Charlson Comorbidity Index; NOS = not otherwise specified.

There were no statistically significant differences in baseline characteristics of the fibrates cohort and the propensity score‐matched control cohort after matching, indicating successful balancing of baseline distribution of covariates between the two cohorts (Table 2).

**TABLE 2:** Baseline characteristics after propensity score matching.

	Fibrates	Propensity‐matched control	p
N	149	298	
Age (mean (SD))	69.42 (7.60)	69.93 (9.31)	0.57
Sex = male N (%)	92 (61.7)	178 (59.7)	0.76
BMI at diagnosis (Mean (SD))	29.1 (5.1)	28.6 (4.9)	0.15
Year of tumour diagnosis N (%)			0.73
2002–2006	21 (14.1)	49 (16.4)	
2007–2011	63 (42.3)	129 (43.3)	
2012–2017	65 (43.6)	120 (40.3)	
CCI (mean (SD))	4.89 (2.75)	4.51 (2.36)	0.13
Smoking status N (%)			0.99
Never	21 (14.1)	43 (14.4)	
Smoker	128 (85.9)	255 (85.6)	
Unknown	0 (0.0)	0 (0.0)	
Histology N (%)			0.56
Adenocarcinoma	107 (71.8)	213 (71.5)	
Large cell	3 (2.0)	8 (2.7)	
Other	4 (2.7)	3 (1.0)	
Squamous	35 (23.5)	74 (24.8)	
Summary stage N (%)			0.76
Localized	68 (45.6)	137 (46.0)	
Regional both	15 (10.1)	34 (11.4)	
Regional extension	13 (8.7)	30 (10.1)	
Regional nodes	16 (10.7)	38 (12.8)	
Regional nos	0 (0.0)	0 (0.0)	
Distant	37 (24.8)	59 (19.8)	
Unstaged	0 (0.0)	0 (0.0)	
Tumour mutation status %			0.77
KRAS	41% (35/85)	37% (64/172)	
EGFR	13% (12/92)	10% (12/121)	
TP53	52% (13/25)	48% (28/59)	

There was no significant difference in the composite primary outcome of cachexia as summarized in Table 3 (49.7% for fibrates cohort vs. 46.6% for control cohort; *p* = 0.56). Among patients with available anthropometric measurements at diagnosis and at 6 months, there was no change in mean BMI at 6 months after diagnosis (28.0 vs. 27.6; *p* = 0.33) nor a significant difference in the mean percent weight change over 6 months (−3.8% vs. − 3.5%; *p =* 0.82).

**TABLE 3:** Cachexia measures.

	Fibrates (*n* = 149)	Propensity matched control (*n* = 298)	*p*
**Cachexia**			
**Composite cachexia N (%)**	**74/149 (49.7)**	**139/298 (46.6)**	**0.56**
Clinically significant weight loss at diagnosis N (%)	36/149 (24.2)	66/298 (22.2)	
> 5% weight loss over 6 months N (%)	48/116 (41.4)	71/167 (42.5)	
> 2% weight loss over 6 months + BMI < 20 N (%)	4/116 (3.4)	6/167 (3.6)	
**BMI and weight loss**			
BMI at 6 months after diagnosis (mean (SD))	28.0 (6.8)	27.6 (6.9)	0.33
% Weight change 6 months after diagnosis (mean [95% CI])	‐ 3.8 [3.1–4.5]	‐ 3.5 [3.1–3.9]	0.82
**Skeletal muscle area analysis**			
**N**	**62**	**101**	
**Male N (%)**	**39 (63%)**	**66 (65%)**	
**Female N (%)**	**23 (37%)**	**35 (35%)**	
Skeletal muscle area (cm^2^) at diagnosis (mean [95% CI])	36.7 [34.5–38.9]	35.7 [33.8–37.7]	
**Male**	**37.2 [34.5–39.9]**	**36.5 [34.3–38.7]**	
**Female**	**35.9 [33.4–38.3]**	**34.2 [31.6–36.8]**	
Skeletal muscle area (cm^2^) at 6 months after diagnosis (mean [95% CI])	35.5 [33.0–38.0]	34.2 [32.2–36.0]	
**Male**	**36.0 [32.9–39.1]**	**35.0 [32.6–37.4]**	
**Female**	**34.6 [32.0–37.2]**	**32.7 [31.5–34.2]**	
% Skeletal muscle area change (mean [95% CI])	‐ 3.3 [3.0–3.6]	‐ 4.2 (3.8–4.6)	**0.03**
**Male**	**−3.2 [2.8–3.6]**	**−4.1 [3.6–4.6]**	
**Female**	**−3.6 [3.1–4.2]**	**−4.4 [3.7–5.0]**	

There were 62 patients (41.6%) within the fibrates cohort and 101 patients within the propensity‐score matched control cohort with CT imaging available for skeletal muscle area measurement. In this population, we found a modest but significant difference in skeletal muscle area over time between the fibrates cohort and the control cohort (−3.3% [3.0–3.6] vs. −4.2% [3.8–4.6]; *p* = 0.03).

**FIGURE 1:**
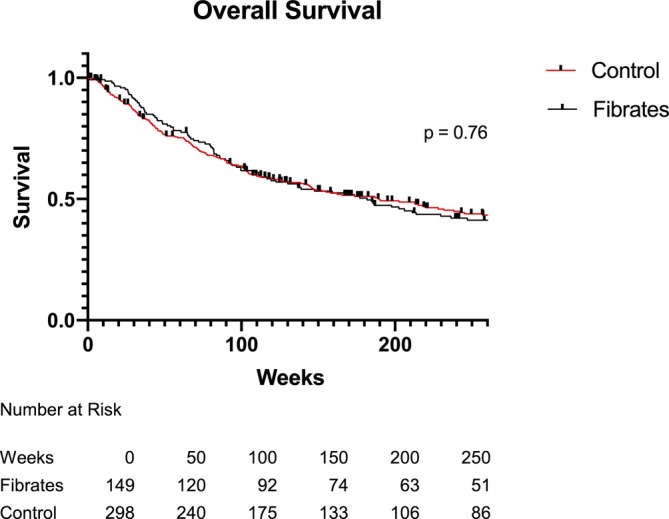
Overall survival.

Kaplan–Meier curves depicting overall survival are presented in Figure 1. Follow‐up data was complete through January 1, 2020. There was no statistically significant difference found between the fibrates cohort and the propensity‐score matched control cohorts (3.5 years vs. 3.6 years; HR 1.04, 95% CI 0.82–1.32).

## 4 Discussion

CACS is a common, debilitating condition that contributes to reduced mobility, worsened quality of life and shortened survival [12, 13, 14]. In preclinical experiments using a genetically engineered mouse model of NSCLC, we have shown that fenofibrate prevents the loss of skeletal muscle mass and body weight. This study sought clinical evidence to validate this finding using retrospective measures of body weight and muscle mass. To our knowledge, this represents the first clinical study that demonstrates a potential protective effect of fibrates for skeletal muscle loss over time.

Cross‐sectional imaging has become the gold standard for quantifying muscle mass in patients with cancer [15]. While there are numerous studies documenting the prognostic implications of low muscle mass at the time of diagnosis, there are very few that track changes in skeletal muscle over time. We have previously shown that CT‐based thoracic skeletal muscle measurement correlates very well with whole‐body skeletal muscle (*R* ^2^ = 0.78), and we used this measure to detect a loss in muscle following chemotherapy in patients with NSCLC [9, 10]. In agreement with our prior data, this study finds that patients with NSCLC tend to lose muscle mass and weight over the initial 6 months after diagnosis, a clinically relevant time span that aligns with standard of care assessments and therapy. The significance of a difference in change in skeletal muscle area of 0.9% between patients on fibrates and the control group is modest but clinically significant as any loss of skeletal muscle has been shown to result in worse clinical outcomes, including decreased tolerance to therapy and decreased overall survival [5]. These results help to highlight the urgent need for therapies that preserve muscle mass during this interval.

We find that the prevalence of the CACS in our population is about 50% using the composite metric. This value agrees well with other groups who have found cachexia in 50% of lung cancer patients at the time of diagnosis and 75% in those with advanced disease [4, 16, 17]. We were unable to confirm previous reports suggesting that *KRAS*‐mutant tumors are associated with cachexia more than other mutations [18, 19]. There is a limited amount of data about the interaction between tumour mutational status and the incidence of CACS in both preclinical and clinical studies, and this study found no significant relationships.

Among patients with NSCLC who were incidentally taking either fenofibrate or gemfibrozil at the time of diagnosis, we found no significant difference between the incidence of CACS compared with a propensity‐score matched control cohort. However, in a subset analysis, patients on fibrates tended to have less skeletal muscle area loss over time as measured on CT imaging compared with the control cohort, suggesting a muscle protective effect of fibrates in the setting of lung cancer. While this finding should be interpreted with caution as it is derived from a secondary analysis and it partly substantiates our findings from a mouse model of lung cancer where the loss of muscle mass was prevented by the use of fenofibrate [7]. In mice, fenofibrate also preserved total body weight but this effect was not observed in the current human study. Unlike humans, mice develop hepatomegaly in response to fibrates and the increase in liver mass significantly contributes to the change in total body weight after the initiation of therapy. Moreover, the population of human subjects is much more heterogeneous than the mouse model and it is possible that fenofibrate may only benefit a subset of patients.

This study found no significant difference in overall survival between the fibrates cohort and the propensity score‐matched control cohort despite the modest protection against muscle wasting; however, this study was likely underpowered to identify differences in overall survival based on the sample size. This result argues against the putative anti‐tumour effects of fibrates that have been suggested in the preclinical literature but have yet to be validated in clinical populations [20].

This study has several strengths, including the moderately large sample size over a 15‐year time period and the use of propensity‐score matched analysis to mitigate the potential for confounding by baseline differences between fibrate‐exposed and fibrate‐unexposed patients. Furthermore, the direct translation of a pre‐clinical model into a retrospective clinical study provides biologic plausibility for the causal effect of fibrates preventing skeletal muscle loss.

As a retrospective study, this study also has several limitations. First, we identified patients based on whether they were recorded to be taking either fenofibrate or gemfibrozil at the time of diagnosis. There was limited information about what amount of time they had been taking the medication prior to diagnosis, which may have affected study outcomes. Additionally, there was significant heterogeneity in both formulations and prescribed doses specifically for fenofibrate making it difficult to draw conclusions specifically about a dose‐dependent relationship with either weight loss or skeletal muscle change. Second, the muscle cross‐sectional area measurements were only performed in a subset of patients who had imaging data available to review, which could potentially suggest some level of either ascertainment or selection bias. For example, patients who potentially had more aggressive or advanced disease would likely have more substantial muscle loss over time and would also likely have necessitated more frequent imaging studies. Third, although all patients received standard of care in terms of cancer‐directed therapy there was limited and heterogeneous information regarding the type and duration of treatments each patient received, which likely affected outcomes.

Future investigation through a prospective study would help validate some of the findings both in this paper as well as the pre‐clinical mouse model by addressing some of the above limitations. Correlating changes in weight and skeletal muscle over time with analyses of blood serum would be another advantage of a prospective study as it could better define the phenotype of patients with CACS and better predict who might best respond to therapy and which patients' treatment might offer meaningful improvements in overall survival and quality of life.

## Conflicts of Interest

Rahmi Elahjji, Tahj Blow, Rahul Grover, Maurice A. Hurd, Bette J. Caan, Elizabeth M. Cespedes Feliciano, Andrew J. Plodkowski, and James H. Flory report no conflicts of interest. Richard F. Dunne received honoraria from Merck and Co. Exelixis Inc. and Incyte for advisory boards and serves on an executive steering committee for Pfizer Inc. Marcus D. Goncalves reports consulting or advisory roles with Scorpion Therapeutics, Skye Biosciences, and Third Arc Bio; stock or other ownership interests in Faeth Therapeutics and Skye Biosciences; patents, royalties and other intellectual property with Weill Cornell Medicine unrelated to the subject of this manuscript.
